# Predicting Major Adverse Carotid Cerebrovascular Events in Patients with Carotid Stenosis: Integrating a Panel of Plasma Protein Biomarkers and Clinical Features—A Pilot Study

**DOI:** 10.3390/jcm13123382

**Published:** 2024-06-09

**Authors:** Hamzah Khan, Abdelrahman Zamzam, Farah Shaikh, Gustavo Saposnik, Muhammad Mamdani, Mohammad Qadura

**Affiliations:** 1 Division of Vascular Surgery, St. Michael’s Hospital, Toronto, ON M5B 1W8, Canada; hamzah.khan@mail.utoronto.ca (H.K.); abdelrahman.zamzam@gmail.com (A.Z.); farah.shaikh@unityhealth.to (F.S.); 2 Department of Surgery, University of Toronto, Toronto, ON M5T 1P5, Canada; 3 Li Ka Shing Knowledge Institute, St. Michael’s Hospital—Unity Health Toronto, Toronto, ON M5B 1W8, Canada; gustavo.saposnik@unityhealth.to (G.S.); muhammad.mamdani@unityhealth.to (M.M.); 4 Division of Neurology, Department of Medicine, University of Toronto, Toronto, ON M5T 1P5, Canada

**Keywords:** carotid stenosis, biomarkers, protein, prognostication, stroke

## Abstract

**Background:** Carotid stenosis (CS) is an atherosclerotic disease of the carotid artery that can lead to devastating cardiovascular outcomes such as stroke, disability, and death. The currently available treatment for CS is medical management through risk reduction, including control of hypertension, diabetes, and/or hypercholesterolemia. Surgical interventions are currently suggested for patients with symptomatic disease with stenosis >50%, where patients have suffered from a carotid-related event such as a cerebrovascular accident, or asymptomatic disease with stenosis >60% if the long-term risk of death is <3%. There is a lack of current plasma protein biomarkers available to predict patients at risk of such adverse events. **Methods:** In this study, we investigated several growth factors and biomarkers of inflammation as potential biomarkers for adverse CS events such as stroke, need for surgical intervention, myocardial infarction, and cardiovascular-related death. In this pilot study, we use a support vector machine (SVM), random forest models, and the following four significantly elevated biomarkers: C-X-C Motif Chemokine Ligand 6 (CXCL6); Interleukin-2 (IL-2); Galectin-9; and angiopoietin-like protein (ANGPTL4). **Results:** Our SVM model best predicted carotid cerebrovascular events with an area under the curve (AUC) of >0.8 and an accuracy of 0.88, demonstrating strong prognostic capability. **Conclusions**: Our SVM model may be used for risk stratification of patients with CS to determine those who may benefit from surgical intervention.

## 1. Introduction

Carotid stenosis (CS) is an atherosclerotic disease characterized by the narrowing of the internal carotid, external carotid, and/or common carotid artery. Atherosclerosis occurs through the initiation of local inflammation induced by increased levels of low-density lipoprotein (LDL) cholesterol, in combination with injury to the endothelium by disease factors such as diabetes mellites, hypertension, and smoking [1,2]. CS can lead to a transient ischemic attack (TIA) or a cerebrovascular attack (CVA), more commonly known as a stroke, and accounts for approximately 7% of all ischemic strokes [3]. Risk factors for CS include hypertension, hypercholesterolemia, diabetes mellitus, and smoking, and with an aging population, the prevalence of CS is on the rise [4]. 

CS can present as either symptomatic, where patients present with a TIA or CVA, or asymptomatic, where patients do not suffer from any symptoms yet can have stenosis with up to 99% reduction in carotid diameter. Surgical interventions, such as carotid endarterectomy (CEA) and carotid artery stenting (CS), are often suggested for patients with symptomatic CS > 50% [5,6]. Additionally, studies such as the Asymptomatic Carotid Atherosclerosis Study (ACAS) trial as well as the Carotid Revascularization Endarterectomy vs. Stenting trial (CREST) suggest that asymptomatic patients with CS > 60% may benefit from CEA, showing a risk reduction of 53% in stroke and death and no significant difference in primary endpoints of stroke, myocardial infarction, or death between asymptomatic patients who received carotid CEA or CS [7,8]. Now, the Society for Vascular Surgery (SVS) suggests that asymptomatic patients with stenosis >60% can be provided CEA only if the 3–5 year perioperative risk of death is <3% [9]. This decision is often made by the judgment of the treating physician based on clinical features alone, such as age, percent stenosis, plaque morphology, past medical history, family history, etc. Current methods used to predict the outcomes following CEA have several limitations and poor performance with AUCs between 0.58 and 0.74 [10], indicating a need for better prediction models. 

Atherosclerosis has clear pathophysiological effects on several cellular pathways, with inflammation and the release of cytokines and growth factors being a significant factor in the initiation and progression of the disease [11,12]. As excessive low-density lipoprotein cholesterol (LDL-C) is taken up by macrophages at the site of arterial injury, the uptake is exhausted, leading to the formation of foam cells. These foam cells not only play a significant role in atherosclerotic plaque formation but also induce the activation of the inflammatory pathway, often associated with their apoptosis and release of inflammatory cytokines such as high sensitivity C-reactive protein (hs-CRP), interleukin 6 (IL-6), as well as tissue necrosis factor (TNF). These cytokines and chemokines recruit and activate Th1 helper T cells at the location of the atherosclerotic plaque that then release Interleukin-2 (IL-2), required for the maturation and activation of T cells, antigen-presenting B cells (which carry CD40 required for their activation), and natural killer cells [13]. This increases the inflammatory environment and increases atherosclerosis in the area. Previous studies have demonstrated an increase in these inflammatory proteins in patients with CS when compared to patients without CS [14,15,16], and hence, patients with CS may also have elevated levels of IL-2 and CD40 [17].

Angiogenesis and inflammation are two key factors in the progression of atherosclerosis that can contribute to the destabilization of atherosclerotic plaques. Hypoxia-inducible factor-1 alpha (HIF-1 α) is a transcription factor known to regulate several genes that are responsible for the homeostasis of oxygen within tissues, specifically in response to hypoxia through glucose, anaerobic respiration, angiogenesis, and inflammation [18]. HIF-1 α has been associated with atheromatous inflammatory plaques, as well as shown to be correlated with CS through its role in angiogenic and proinflammatory pathways in human and murine models [19,20]. Hence, in this study, we investigated inflammatory and angiogenic proteins in this pathway, specifically C-X-C Motif Chemokine Ligands (CXCL), galectin proteins, as well as growth factors such as angiopoietin-related proteins. Bone morphogenic proteins also play an important role in angiogenesis, regulating the expression of various angiogenic factors, such as vascular endothelial growth factor (VEGF), as well as stimulating the proliferation and migration of endothelial cells [21]. Investigating these proteins and pathways may allow for discovering novel prognostic biomarkers for adverse cardiovascular outcomes in patients with CS. 

There is a lack of a strong clinically relevant biomarker or a combination of biomarkers that can predict adverse outcomes in this patient population. In this pilot study, we investigated several inflammatory proteins and growth factors, their association with CS, and their ability to predict adverse outcomes, including TIA, CVA, and death. CS often goes undetected before catastrophic adverse events occur; hence, having the capability of predicting those patients who are at a higher risk of an adverse event is crucial in devising targeted and effective preventive strategies.

## 2. Materials and Methods

### 2.1. Patient Selection

Asymptomatic patients (no cerebrovascular symptoms, including transient ischemic attack (TIA), stroke, or other carotid-related ischemic events within the past six months) attending St. Michael’s Hospital clinics between 2018 and 2020 for carotid artery ultrasound were recruited. All patients underwent a thorough physical examination by a vascular specialist and had a carotid Doppler ultrasound conducted by a vascular ultrasound technician. Percent stenosis of the carotid artery was determined based on the Washington criteria for CS [22,23]. Patients with non-CS-related strokes were excluded from this study. To ensure accuracy, two vascular surgeons and one neurologist reviewed each carotid patient’s ultrasound to determine the degree of CS. Additionally, every stroke patient underwent comprehensive evaluation by two vascular surgeons and a neurologist, including advanced imaging (CTA, MRI, and US) to confirm the carotid artery’s involvement. Based on the internal carotid artery (ICA) peak systolic velocity (PSV), end-diastolic velocity (EDV), and the ratio of the ICA to common carotid artery (CCA) PSVs, patients were determined to have either <50% stenosis (hemodynamically non-significant CS, the “<50% CS” group), ≥50% stenosis (“CS” group). Patients with CS < 50% stenosis were considered as hemodynamically non-significant stenosis, as this population of patients is not surgically treated for CS by specialists. Patients with only total occlusions within the carotid artery were excluded due to the low risk of embolic stroke from the occluded carotid artery. 

### 2.2. Baseline Characteristics and Cardiovascular Risk Factors

The patient’s past medical history, including the history of hypertension, hypercholesterolemia, diabetes mellitus, smoking status, history of stroke, transient ischemic attacks (TIA), coronary artery disease, congestive heart failure, and renal insufficiency, were collected. Patients taking lipid-lowering therapy or individuals with total cholesterol levels greater than 5.2 mmol/L or triglyceride levels greater than 1.7 mmol/L were considered hyperlipidemic. A systolic blood pressure > 130 mm Hg or diastolic blood pressure > 80 mm Hg was considered hypertensive. Patients with glycosylated hemoglobin A1c > 6.5% were considered diabetic.

### 2.3. Sample Collection

Blood was drawn from the antecubital vein into citrated vacutainer tubes. Plasma was isolated by centrifugation at 1000× *g* for 10 min at 4 °C and stored at −80 °C until protein quantification. Luminex Discovery Assay Kits from Bio-Techne R&D Systems (LXSAHM-15, Minneapolis, MN, USA) were used as described by the manufacturer to quantify the levels of the following inflammatory proteins: C-X-C Motif Chemokine Ligands 1 (CXCL1); CXCL6, Galectin-1; Galectin-9; interleukin2-(IL-2); Cluster of differentiation 40 (CD40); CD40 ligand (CD40L), and the following growth factors: angiopoietin-1; angiopoietin-like protein 3 (ANGPTL3); ANGPTL4; ANGPTL6; and bone morphogenetic protein 2 (BMP-2). Each patient provided one blood sample, and each sample’s protein quantification was run in duplicates, and values were averaged between the two runs. 

### 2.4. Prospective Follow-Up

Patients were followed up for 24 months at 6 or 12-month intervals, according to the SVS guidelines [2,9]. At each visit, the patient underwent a repeat carotid Doppler ultrasound to determine the degree of CS, and each patient had an examination by a vascular specialist. Any changes in clinical history or medication were recorded through interviews with the patients and reviews of the patients’ medical charts. Any amaurosis fungus, TIA, strokes, myocardial infarctions (MI), carotid surgical interventions, including CEA and carotid stenting, and cardiovascular-related death were noted. No patients were lost to follow-up.

### 2.5. Outcomes

The primary outcomes for this study included any incidence of major adverse cerebrovascular and carotid-related events (MACCE), defined as the composite of the incidence of amaurosis fugax, TIA, or CVA, and any carotid-related surgical intervention, such as CEA or carotid stenting.

### 2.6. Statistical Analysis

In this study, we summarized demographic and clinical characteristics by presenting means and standard deviations for continuous variables and frequencies and percentages for categorical variables. Initial group comparisons were conducted using the Mann–Whitney test for continuous variables and chi-square tests for categorical variables. Subsequently, we identified significant proteins for further investigation in a protein panel model. 

For modeling, the dataset was split into a 70% training set and a 30% testing set. The study model was constructed using support vector machine (SVM) regression. SVM is an algorithm used for classification tasks. The coefficients from the linear SVM represent the weights assigned to each feature, which is used to make predictions. We developed three distinct models to assess risk prediction. The first model incorporated the significant proteins identified through our comparative analysis (protein panel model). The second model comprised clinical features, including Age (years), Sex (Male = 1; Female = 0), Hypertension (Yes = 1; No = 0), Dyslipidemia (Yes = 1; No = 0), Diabetes (Yes = 1; No = 0), Smoking (0.19 × Current = 2; Past = 1; No = 0), congestive heart failure (CHF) (Yes = 1; No = 0), and coronary artery disease (CAD) (Yes = 1; No = 0) (clinical features model). Lastly, the third model combined both the significant proteins and clinical features (full model). To assess the comparative performance of the models, we conducted DeLong’s test to examine statistical differences in the Area Under the Receiver Operating Characteristic curves (AUCs). We also used a range of evaluation metrics to comprehensively evaluate the models’ effectiveness in diagnosing CS and predicting 2-year Major Adverse Carotid-Related Cerebrovascular Events (MACCE). These metrics included Sensitivity, Specificity, Positive Predictive Value (PPV), Negative Predictive Value (NPV), and Accuracy. For Kaplan–Meier analysis, each patient was assigned a specific score based on their protein concentration, leading to the division of the overall cohort into high and low-score groups. Event-free survival curves were computed, and event-free survival between subgroups was compared using the log-rank test. A linear SVM was used for prognostic modeling. The linear SVM finds the optimal hyperplane that maximizes the margin between the classes. The coefficients from the linear SVM represent the weights assigned to each feature. These coefficients were used in a linear equation to model the relationship between the features and the MACCE. Statistical significance was established at *p* < 0.05 (two-sided), and these analyses were conducted using Prism Version 10.1.0 and Python V3.

## 3. Results

### 3.1. Patient Demographics

A total of 155 patients with CS were enrolled, alongside 94 patients exhibiting hemodynamically non-significant CS (<50% stenosis) who served as the <50% CS control group (Table 1). Males comprised 63% of the overall population, with the CS group displaying a significantly higher age compared to the <50% CS group (72.8 ± 8.61 years vs. 66.7 ± 10.3 years, respectively). Furthermore, the CS group exhibited notably higher rates of hypercholesterolemia and diabetes mellitus and a tendency toward increased rates of coronary artery disease. Conversely, no significant differences were observed in terms of sex, hypertension, smoking status, and congestive heart failure between the two groups.

### 3.2. Plasma Levels of Inflammatory Proteins and Growth Factors

**Figure 1:**
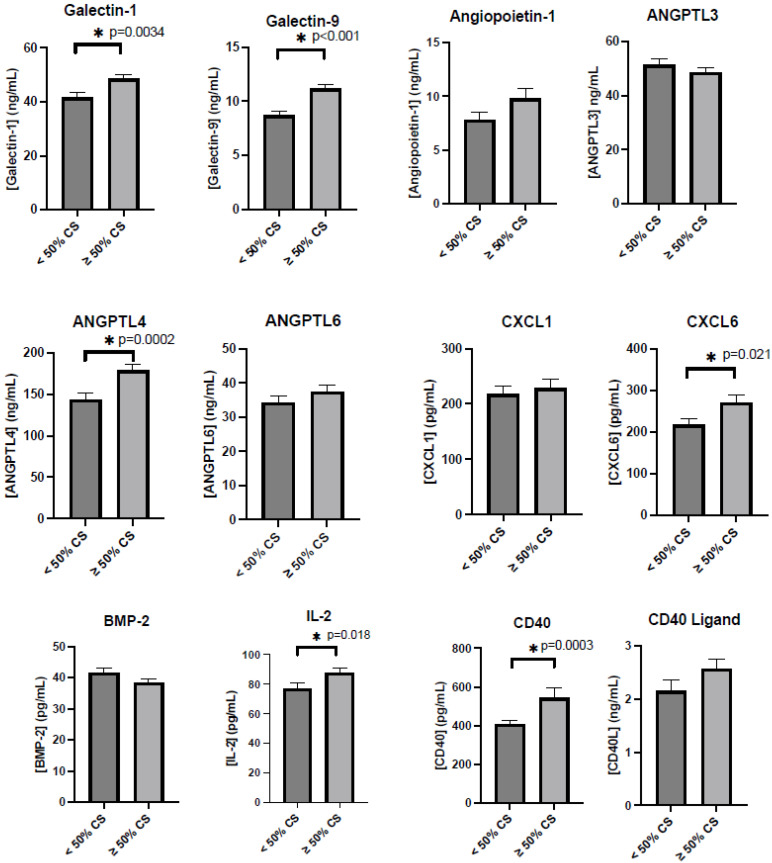
Levels of inflammatory proteins and growth factors in patients with and without carotid stenosis (CS). CS was defined as those with stenosis in the internal, external, or common carotid artery of >50%. C-X-C Motif Chemokine Ligand 6 (CXCL6), Interleukin-2 (IL-2), Angiopoietin-like 4 (ANGPTL4), Cluster of differentiation 40 (CD40), CD40 ligand (CD40L), bone morphogenetic protein 2 (BMP-2). * Represents a significant difference between patients with carotid stenosis <50% and those with carotid stenosis >50%, with a *p* value < 0.05.

In this study, patients with CS exhibited notably higher plasma concentrations of several biomarkers when compared to patients with <50% stenosis. Specifically, the median plasma levels of CXCL6 were 186.3 pg/mL [IQR 148.0–299.9] in the CS group vs. 176.7 pg/mL [IQR 134.8–255.1] in the <50% CS group, with a statistical significance of *p* = 0.043. For IL-2, the median was 83.7 pg/mL [IQR 54.6–114.3] compared to 66.6 pg/mL [IQR 45.6–106.7]; *p* = 0.027. Galectin-1 levels were 45 ng/mL [IQR 25–60] against 39.7 ng/mL [IQR 28.1–50.7]; *p* = 0.006. Galectin-9 levels were markedly higher at 10.3 ng/mL [IQR 7.8–13.3] compared to 8.5 ng/mL [IQR 5.9–10.8]; *p* = 0.001. ANGPTL4 levels also differed significantly, with the CS group having a median of 157.2 ng/mL [IQR 116.3–218.4] vs. 130.6 ng/mL [IQR 102.0–162.7] in the <50% group; *p* = 0.002. There were no statistically significant differences in the levels of angiopoietin-1, ANGPTL3, ANGPTL6, CXCL1, BMP-2, and CD40L between the two groups, as illustrated in Figure 1.

### 3.3. Predicting Major Adverse Events

Follow-up data were available for an average duration of 22 ± 5 months, with the majority (92%) of the cohort having complete data for the entire 24-month follow-up period. Within the group with <50% CS, there were only four incidents, all of which were myocardial infarctions (MIs). Conversely, in the group with more significant CS, there were 10 instances requiring surgical intervention, along with seven MIs and six strokes reported. Statistical analysis revealed a significantly elevated incidence of Major Adverse Carotid-related Cerebrovascular Events (MACCE) in the CS group as opposed to the <50% CS group (*p* = 0.034), as detailed in Table 2.

This study evaluated the prognostic potential of specific plasma proteins in patients with CS using support vector machine (SVM) models. These models, which were formulated based on the plasma levels of CXCL6, IL-2, Galectin-9, and ANGPTL4—proteins found to be significantly higher in CS patients—served to assess the likelihood of Major Adverse Carotid Cerebrovascular Events (MACCE). Galectin-1 was excluded from this model due to its substantial correlation with Galectin-9, exhibiting a correlation coefficient greater than 0.7. CD40 was excluded from this model due to its low feature importance and negligible change in the model’s overall predictive capability. 

**Figure 2:**
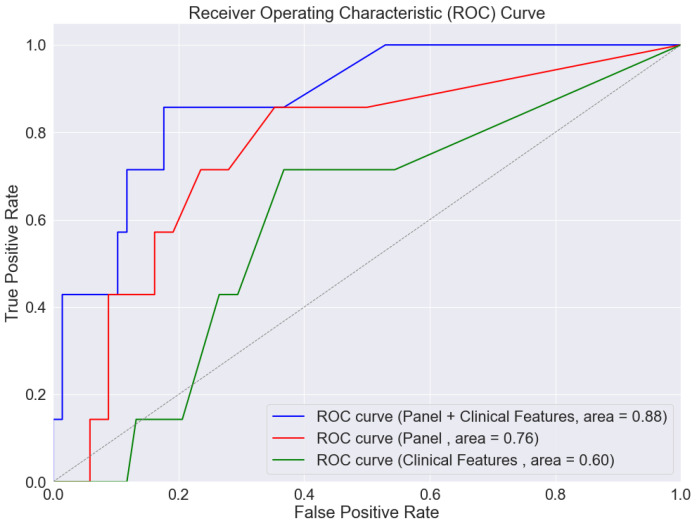
Receiver operating characteristics (ROC) curve demonstrating prognostic capability of support vector machine models in predicting major adverse carotid cerebrovascular events (MACCE), which is defined as the composite of stroke, carotid surgical interventions, myocardial infarctions, and cardiovascular-related death. The green line represents a model that includes clinical features only: age; sex; hypertension; hypercholesterolemia; diabetes mellitus; smoking status; and history of congestive heart failure or coronary artery disease. The red line represents a model, including the plasma protein levels of C-X-C Motif Chemokine Ligand 6 (CXCL6), Interleukin-2 (IL-2), angiopoietin-like 4 (ANGPTL4), and Galectin-9. The blue line represents a model that includes both clinical features and plasma protein levels. Area under the curve (AUC).

The accuracy of the SVM models was measured by their ability to predict MACCE. Models using only clinical features demonstrated limited predictive accuracy (as shown in Figure 2, green line, with an Area Under the Curve (AUC) of 0.60). The accuracy was notably better when utilizing the biomarker panel alone (Figure 2, red line; AUC = 0.76). However, the integration of both clinical features and the biomarker panel into the predictive model substantially enhanced its accuracy, achieving an AUC of 0.88, a sensitivity of 0.7, and a specificity of 0.92 (Figure 2, blue line, and Table 3). 

**Figure 3:**
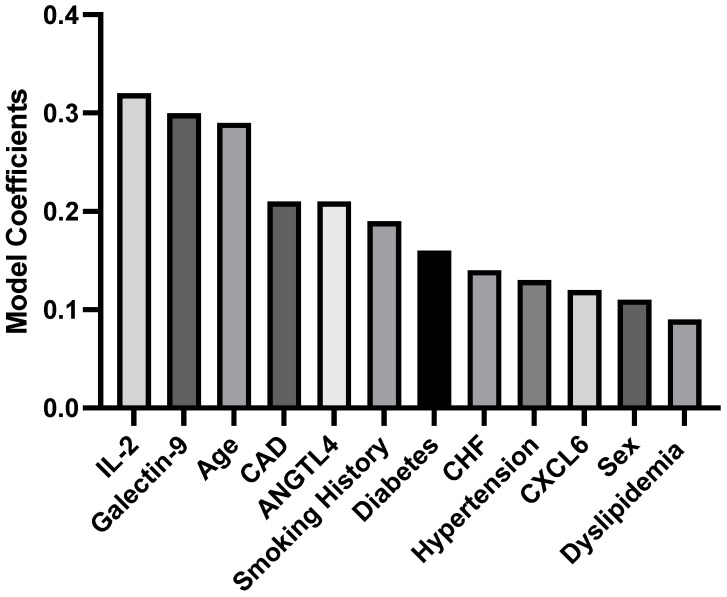
Model coefficients of clinical features and plasma proteins.

Furthermore, the model coefficients were used to devise an equation, illustrated in Figure 3, that calculates the two-year probability of MACCE occurrence in patients by integrating both clinical features and normalized levels of the four plasma proteins.

### 3.4. Probability Score Analysis

Probability of MACCE in the next two years = 0.29 × Age (years) + 0.11 × Sex (Male = 1; Female = 0) + 0.13 × Hypertension (Yes = 1; No = 0) + 0.09 × Dyslipidemia (Yes = 1; No = 0) + 0.16 × Diabetes (Yes = 1; No = 0) + 0.19 × Smoking (Current = 2; Past = 1; No = 0) + 0.14 × CHF (Yes = 1; No = 0) + 0.21 × CAD (Yes = 1; No = 0) + 0.12 × CXCL6 (normalized value pg/mL) + 0.32 × IL-2 (normalized value pg/mL) + 0.21 × ANGPTL4 (normalized value pg/mL) + 0.30 × Galectin-9 (normalized value pg/mL)

The probability of having MACCE in the next two years = 0.29 × Age (years) + 0.11 × Sex (Male = 1; Female =) + 0.13 × Hypertension (Yes = 1; No = 0) + 0.09 × Dyslipidemia (Yes = 1; No = 0) + 0.16 × Diabetes (Yes = 1; No = 0) + 0.19 × Smoking (Current = 2; Past = 1; No = 0) + 0.14 × CHF (Yes = 1; No = 0) + 0.21 × CAD (Yes = 1; No = 0) + 0.12 × CXCL6 (normalized value pg/mL) + 0.32 × IL-2 (normalized value pg/mL) + 0.21× ANGPTL4 (normalized value pg/mL) + 0.30 × Galectin-9 (normalized value pg/mL) (1)

### 3.5. Risk Stratification of Patients Based on Clinical Features and Protein Biomarker Panel

In the following phase, we assessed the effectiveness of the previously derived equation in accurately identifying patients at higher risk of MACCE by considering both their biomarker profiles and clinical characteristics. Utilizing Equation (1), we computed probability scores for each patient, which were subsequently used to categorize individuals into “High” or “Low” risk groups based on a probability score threshold determined through receiver operative characteristics analysis. 

**Figure 4:**
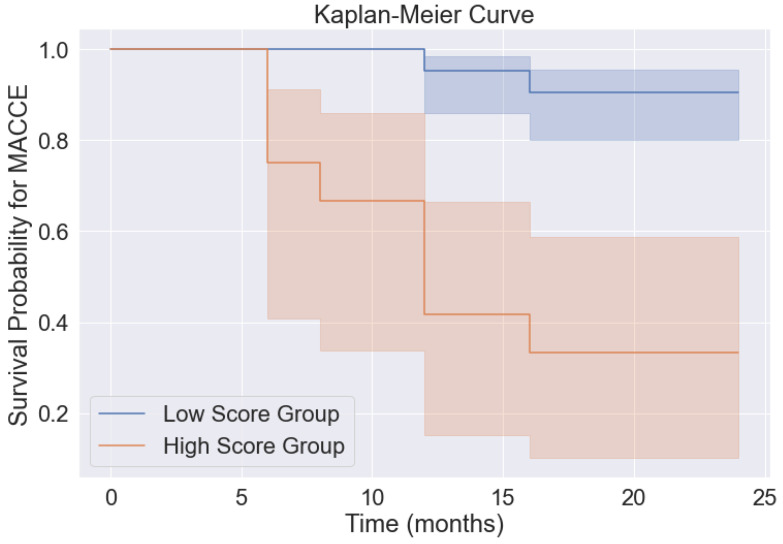
Kaplan–Meier analysis representing MACCE survival over a period of 24 months between patients with high and low probability scores. Scores were calculated using the probability equation, and patients were then split into high vs. low based on cut-off values obtained by receiver operating characteristics analysis. Shaded regions represent 95% Confidence Intervals.

Kaplan–Meier analysis unveiled a significant distinction between these risk groups. Patients with higher probability scores exhibited a markedly increased likelihood of experiencing MACCE events compared to their counterparts with lower scores (Log-rank value = 30.9; *p*-value < 0.001) (see Figure 4). Specifically, in the low-score group, survival probabilities at 12 and 24 months stood at 95% and 90%, respectively. Conversely, in the high-score group, survival probabilities at the same time intervals were notably lower, measuring 42% and 33%, respectively (see Figure 4).

## 4. Discussion

In this pilot study, we were able to demonstrate that four plasma proteins could be combined using computational modeling to diagnose CS (see Appendix A) and predict adverse outcomes in patients with CS. Specifically, when CXCL6, IL-2, ANGPTL4, and Galectin-9 plasma concentrations were used in combination with clinical features such as hypertension, dyslipidemia, and age, our models could predict MACEE with AUCs of 0.88, which represented good prognostic capability [24]. 

Several inflammatory proteins and growth factors were investigated for their relationship with CS and their ability to predict adverse outcomes, such as stroke, myocardial infarction, and the need for surgical intervention. Inflammatory and endothelial growth factor families, including C-X-C Motif Chemokine Ligands (CXCL), galectin proteins, and interleukins, as well as growth factors, such as angiopoietin-related proteins and bone morphogenic proteins, have demonstrated their association with CS [25,26,27,28]. The CXCL family are small proteins involved in inflammation and the immune response, with functions including neutrophil and macrophage attraction, by CXCL1. Macrophages up-take oxidatively modified LDL within atherosclerotic plaques, leading to an inflammatory response and recruitment of inflammatory cells that released interleukins such as IL-2, leading to the progression of the disease [29]. Cell permeability, proliferation, and apoptosis after ischemia-reperfusion injury through the HIF-1 α pathway are regulated by CXCL6. CXCL1 and CXCL6 have not been investigated in CS previously [30,31,32]. It was evident in our study that patients with CS had significantly elevated levels of CXCL6 and IL-2 but not CXCL1 compared to controls.

Similarly, galectins are a family of proteins that have a wide range of functions, including the regulation of inflammation and immunity, the activation of macrophages, and cellular proliferation [33]. They bind to glycosylated protein receptors to activate or inhibit inflammatory and immune responses [34]. Galectin-1 is an evolutionarily conserved β-galactoside-binding lectin that functions by reducing the synthesis of proinflammatory cytokines [35]. Contrarily, Galectin-9, in the same family, has the opposite effect by promoting the recruitment of monocytes to areas of inflammation. Galectin 9 has been previously demonstrated to facilitate leukocyte recruitment in patients with peripheral arterial disease [36]. Patients with diabetes often have elevated levels of glycosylated proteins, which is another known contributor to the progression of atherosclerotic disease [37]. Cancer models have demonstrated that CXCLs and Galectins are regulated through the HIF-1 α pathway [38,39]. This pathway has also been implicated in atherosclerotic disease in response to ischemia; hence, these proteins were investigated as potential prognostic biomarkers of CS. Both galectin-1 and galectin-9 were significantly elevated in patients with CS in this study. 

Lastly, atherosclerotic disease induces the release of several growth factors, forcing the differentiation of muscle cells, proliferation of immune cells, and the growth of new tissue and vessels in order to cope with the pathophysiological changes at the location of the plaque growth [40]. Angiopoietin-1 has been implicated in CS, demonstrating lower levels in patients with unstable plaques [27]. The Angiopoietin-like protein (ANGPTL) family of proteins has a structural homology to angiopoietins. They also play important roles in the regulation of lipid metabolism and angiogenesis [41]. ANGPTL3, ANGPTL4, and ANGPTL6 are related proteins associated with lipoprotein metabolism and angiogenesis [42]. Bone morphogenic proteins are known to play a role in angiogenesis by regulating angiogenic factors such as vascular endothelial growth factor (VEGF) and stimulating the proliferation and migration of endothelial cells [21]. BMPs have not previously been implicated in CS, but we investigated BMP-2 in this study, as it has been previously demonstrated to increase atherosclerotic burn in patients with coronary artery disease and DM [43]. The mentioned proteins are implicated in pathways known to be associated with the progression of atherosclerotic disease and, hence, were chosen for investigation as potential prognostic biomarkers for CS. Of these proteins, only ANGPTL4 was found to be significantly elevated in patients with CS in this study. 

The current gold standard for the diagnosis of CS is through Doppler ultrasounds of the carotid arteries [9]. In this study, the biomarkers we suggested had a good capability of diagnosing patients with CS without the need for ultrasound. Though in most clinical settings, ultrasound for the diagnosis of CS would be the first-line diagnostic tool, the method suggested in this manuscript needs to be further validated in larger clinical trials prior to its use in a diagnostic setting. We obtained an AUC of 0.82 for these biomarkers in combination with clinical features, which suggests that these biomarkers are associated with CS. 

The treatment options for asymptomatic CS have been debated; however, recent studies and guidelines provided by SVS suggest that CEA should be provided to asymptomatic CS patients only if the 3–5 year post-operative risk of stroke and death is <3% [8,9]. Although our data need to be validated in a larger cohort, our suggested model utilizing a biomarker panel demonstrates that a panel of protein biomarkers could be used in combination with clinical features to allow for the prediction of the probability of MACCE within the following two years. This may guide healthcare providers’ care for patients with CS as well as allow for personalized risk stratification. This method can not only allow for risk stratification for surgical interventions but also for the detection of asymptomatic patients who are at a higher risk of adverse events, giving physicians the option of providing those patients with more aggressive medical management for the prevention of CS-related adverse events. 

This is the first study that we know of that has used a novel panel of plasma protein biomarkers that are related to the atherosclerotic process in combination with clinical features of patients to predict MACCE in patients with asymptomatic CS. Our SVM model was able to predict MACCE with a strong accuracy of 0.89. Other models have demonstrated sub-optimal performances with AUCs between 0.54 and 0.74 [44]. Other studies have been conducted to predict outcomes in patients with CS [45]; however, most investigated models for adverse cardiovascular events post-surgical intervention; however, we have demonstrated a novel model that can predict MACCE in patients who neither have symptoms nor have undergone surgical procedures, making it valuable for use in an outpatient clinical setting.

Computational models, such as random forest models and support vector machine models, have become increasingly accurate for diagnostic and prognostic modeling in the medical field. Both methods prevent overfitting and can provide strong generalizability. However, this is still a relatively new field, with advanced computational models not being highly adopted into clinical care [46]. In combination with both the clinical features and a plasma biomarker panel, our models for diagnosis and predictions of MACE and MACCE can give physicians an insight into the probabilities of these events occurring in their patients and allow for evidence-based decision-making. We have proposed a simple equation that could be used as a “clinical calculator” to risk-stratify patients with CS and to decide if surgical treatment would be beneficial; however, this calculator needs to be validated in a larger cohort of patients. 

There are several limitations to our study. As this was a pilot study, our sample size was relatively low. A second limitation was that atrial fibrillation was not considered in the clinical characteristics when initial baseline measurements were obtained. To confirm the accuracy and generalizability of our model, a larger sample size will be required. CS is a chronic disease, and longer follow-up is needed in future studies to validate our proposed protein biomarker panel. Lastly, conducting numerous comparisons is linked to the potential for false significance.

## 5. Conclusions

In conclusion, we have proposed a model that included a four-protein biomarker panel and predictive models that may be used for both diagnostic and prognostic biomarkers for CS. This study demonstrates early results in the capability of using plasma biomarkers in combination with clinical risk factors for the risk stratification of patients with CS. These models may help vascular specialists decide which patients should be offered surgical management, such as CEA or carotid stenting, and which may benefit more from conservative management.

## Figures and Tables

**Table 1:** Demographics and clinical features of patients recruited with and without carotid stenosis (CS).

		<50% CS	≥50% CS	*p*
		(n = 94)	(n = 155)	
		Mean ± SD		
Age		66.7 ± 10.3	72.8 ± 8.61	0.001
Sex	Male	59 (63)	99 (63)	0.861
	Female	35 (37)	56 (37)	
		% (n)		
Hypertension		65 (69)	116 (75)	0.328
Hyperlipidemia		61 (65)	134 (86)	<0.001
DM		16 (17)	49 (32)	0.011
Smoking		64 (68)	115 (74)	0.288
CHF		3 (3)	5 (3)	0.988
CAD		27 (29)	63 (41)	0.058
Statin		73 (69)	89 (138)	0.0026
ACEi/ARB		50 (47)	89 (57)	0.2939
B-bl		27 (29)	32 (50)	0.5752
CCB		18 (17)	35 (23)	0.4258
Renal Insufficiency		0 (0)	0(0)	>0.999
Diuretic		11(12)	21 (14)	0.8454
Oral Hypoglycemic		12 (13)	29 (18)	0.2903
Insulin		1 (1)	12 (8)	0.0204
Antiplatelet(s) Only		57 (54)	74 (114)	0.0118
Anticoagulant(s) Only		6 (6)	8 (12)	0.8037
Combination of Antiplatelet + Anticoagulant		4 (4)	5 (7)	0.9999

DM, Diabetes mellitus; CHF, Congestive Heart Failure; CAD, Coronary Artery Disease; ACEi/ARB, Angiotensin-Converting Enzyme Inhibitors; CCB, Calcium Channel Blocker; CS, Carotid Stenosis. Continuous variables are presented as mean ± standard deviation, and categorical variables are presented as % (n), where n represents the total number of patients.

**Table 2:** Distribution of adverse events during the 24-month follow-up period in patients with and without CS.

	<50% CS (n = 94)	CS (n = 155)	*p*
Surgical Intervention	0 (0)	10 (7)	0.012
MI	4 (4)	7 (5)	0.923
Stroke	0 (0)	6 (4)	0.053
MACCE	4 (4)	19 (12)	0.034

MI, Myocardial Infarction; MACCE, Major Adverse Carotid-related Cerebrovascular Events; CS, Carotid Stenosis. Data are presented as n(%), where n represents the number of patients with overall percentage in brackets.

**Table 3:** Model evaluation metrics for SVM model to predict major adverse carotid cerebrovascular events.

	AUC	Accuracy	Sensitivity	Specificity	F1 Score
Panel	0.76	0.80	0.65	0.85	0.62
Clinical Feature	0.60	0.70	0.50	0.75	0.47
Panel + Clinical Feature	0.88	0.88	0.70	0.92	0.72

**Figure A1:**
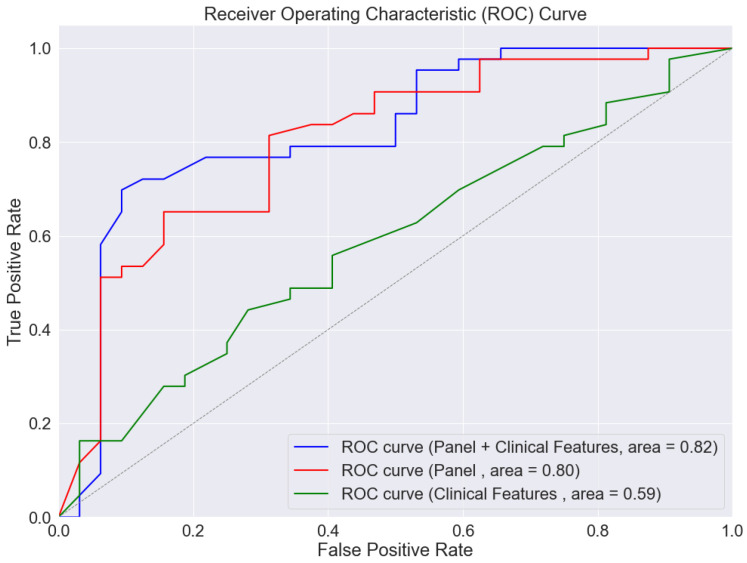
Receiver operating characteristics (ROC) curve demonstrating diagnostic capability of three random forest models. The green line represents a model that includes clinical features only, age, sex, hypertension, hypercholesterolemia, diabetes mellitus, smoking status, and history of congestive heart failure or coronary artery disease. The red line represents a model, including the plasma protein levels of C-X-C Motif Chemokine Ligand 6 (CXCL6), Interleukin-2 (IL-2), angiopoietin-like 4 (ANGPTL4), and Galectin-9. The blue line represents a model that includes both clinical features and plasma protein levels. Area under the curve (AUC).

**Figure A2:**
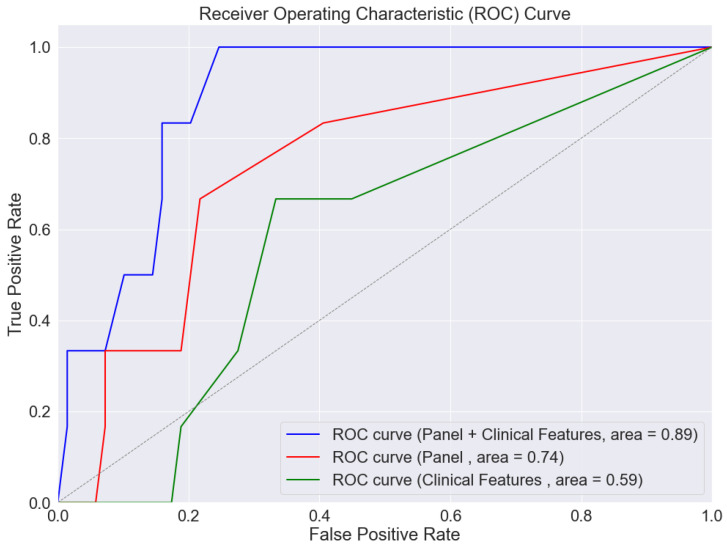
Receiver operating characteristics (ROC) curve demonstrating prognostic capability of three random forest models in predicting major adverse cardiovascular events (MACE), defined as the composite of stroke, myocardial infarctions, and cardiovascular-related death. The green line represents a model that includes clinical features only, age, sex, hypertension, hypercholesterolemia, diabetes mellitus, smoking status, and history of congestive heart failure or coronary artery disease. The red line represents a model including the plasma protein levels of C-X-C Motif Chemokine Ligand 6 (CXCL6), Interleukin-2 (IL-2), angiopoietin-like 4 (ANGPTL4), and Galectin-9. The blue line represents a model that includes both clinical features and plasma protein levels. Area under the curve (AUC).

## Data Availability

Data are available from authors upon request.

## Appendix A

### Appendix A.1. Diagnostic Capabilities of Plasma Proteins

To predict the diagnostic capability of the four elevated plasma proteins, CXCL6, IL-2, Galectin-9, and ANGPTL4, three random forest predictive models were created. For RF, multiple decision trees are constructed. RF generates feature importances, which indicate the contribution of each feature to the model’s predictive power. The first model was created based on using solely clinical features to distinguish the CS from the <50% CS group (Figure A1, Green). With these values, the model’s ability to predict those with CS was low, with an area under the curve (AUC) of 0.59. The second model created using the four plasma proteins demonstrated a good diagnostic capability with an AUC of 0.8 (Figure A1, Red). Creating a final model combining both the clinical features with the four plasma proteins demonstrated further improved diagnostic capability, with an AUC of 0.82 and an accuracy of 0.81 (Figure A1, Blue, and Table A1).

**Table A1:** Model evaluation metrics for random forest model to diagnose carotid artery stenosis.

	AUC	Accuracy	Sensitivity	Specificity	F1 Score
Panel	0.80	0.78	0.65	0.82	0.66
Clinical Feature	0.59	0.68	0.55	0.70	0.52
Panel + Clinical Feature	0.82	0.81	0.72	0.85	0.70

### Appendix A.2. Diagnostic Capabilities of Plasma Proteins

In the first model, with clinical features alone, the model could predict MACE with an AUC of 0.56. The model created with the four proteins only increased the accuracy of the model (AUC = 0.74); however, combining both clinical features in combination with the four proteins allowed for the highest accuracy of predicting MACE, with an AUC of 0.89 and accuracy of 0.85 (Figure A2 and Table A2).

**Table A2:** Model evaluation metrics for random forest model to predict major adverse cardiovascular events (MACE).

	AUC	Accuracy	Sensitivity	Specificity	F1 Score
Panel	0.74	0.70	0.68	0.72	0.68
Clinical Feature	0.59	0.60	0.55	0.65	0.56
Panel + Clinical Feature	0.89	0.85	0.80	0.90	0.81
